# Calour: an Interactive, Microbe-Centric Analysis Tool

**DOI:** 10.1128/mSystems.00269-18

**Published:** 2019-01-29

**Authors:** Zhenjiang Zech Xu, Amnon Amir, Jon Sanders, Qiyun Zhu, James T. Morton, Molly C. Bletz, Anupriya Tripathi, Shi Huang, Daniel McDonald, Lingjing Jiang, Rob Knight

**Affiliations:** a State Key Laboratory of Food Science and Technology, Nanchang University, Nanchang, China; b Department of Pediatrics, University of California San Diego, La Jolla, California, USA; c Department of Biology, University of Massachusetts Boston, Boston, Massachusetts, USA; d Department of Computer Science and Engineering, University of California San Diego, La Jolla, California, USA; e Department of Family Medicine and Public Health, University of California San Diego, La Jolla, California, USA; f Sheba Medical Center, Ramat Gan, Israel; g Center for Microbiome Innovation, University of California San Diego, San Diego, California, USA; University of Trento

**Keywords:** analysis, contamination, heatmap, microbiome, visualization

## Abstract

Microbiome analyses can be challenging because microbial strains are numerous, and often, confounding factors in the data set are also numerous. Many tools reduce, summarize, and visualize these high-dimensional data to provide insight at the community level. However, they lose the detailed information about each taxon and can be misleading (for example, the well-known horseshoe effect in ordination plots). Thus, multiple methods at different levels of resolution are required to capture the full range of microbial patterns. Here we present Calour, a user-friendly data exploration tool for microbiome analyses. Calour provides a study-centric data model to store and manipulate sample-by-feature tables (with features typically being operational taxonomic units) and their associated metadata. It generates an interactive heatmap, allowing visualization of microbial patterns and exploration using microbial knowledge databases. We demonstrate the use of Calour by exploring publicly available data sets, including the gut and skin microbiota of habitat-switched fire salamander larvae, gut microbiota of Trichuris muris-infected mice, skin microbiota of different human body sites, gut microbiota of various ant species, and a metabolome study of mice exposed to intermittent hypoxia and hypercapnia. In these cases, Calour reveals novel patterns and potential contaminants of subgroups of microbes that are otherwise hard to find. Calour is open source under the Berkeley Software Distribution (BSD) license and available from https://github.com/biocore/calour.

**IMPORTANCE** Calour allows us to identify interesting microbial patterns and generate novel biological hypotheses by interactively inspecting microbiome studies and incorporating annotation databases and convenient statistical tools. Calour can be used as a first-step tool for microbiome data exploration.

## Abstract (precis)

Calour allows us to identify interesting microbial patterns and generate novel biological hypotheses by interactively inspecting microbiome studies and incorporating annotation databases and convenient statistical tools. Calour can be used as a first-step tool for microbiome data exploration.

## INTRODUCTION

Recent advances in next-generation sequencing technology have allowed us to study microbial communities without cultivating individual microbes. There has been growing interest in microbiome research due to the fact that microorganisms are ubiquitous and fundamental players in both environmental and human health. Thus, it is important to profile microbial communities taxonomically and functionally in order to understand where they are and what they are doing.

Amplicon sequencing of marker genes, such as 16S rRNA, 18S rRNA, and ITS (internal transcribed spacer), has been successfully applied to identify the taxa present in microbial communities. Traditionally, amplicon sequences are clustered into operational taxonomic units (OTUs) at a given sequence identity level. This overcomes sequencing errors, but the ability to distinguish closely related taxa is lost. Recently, methods have been developed to infer the true amplicon sequences (also known as sub-OTU or sOTU) and their abundances at single-nucleotide resolution (1–3). Thus, each taxon can be represented by a unique amplicon sequence on defined genomic regions (such as the V4 region of 16S rRNA genes), making them directly comparable across studies. In fact, several databases have been developed (spongeEMP [4] and IJSEM phenotypic database [5]) or are now under development (dbBact [http://dbbact.org/main] and redbiom [https://github.com/biocore/redbiom]) to annotate amplicon sequences, enabling accumulation of information from multiple experiments about the bacterial sequences. Such annotations may include, for example, whether it is a known contaminant, what samples have been seen to contain it, or what interesting biological conclusions are associated with it. Combining information from these databases into the analysis of a given experiment can enhance the biological interpretation of the results.

In microbiome studies, a two-dimensional sample-by-feature table is typically generated, where features are often OTUs or sOTUs. Each value in the table is the relative abundance of a feature for a given sample. There are usually hundreds or thousands of samples and features in the table. The large number of samples and high dimensionality of the data make it difficult to interpret without proper statistical and visualization tools. Multiple “summary methods” have been developed to summarize the data (alpha diversity, beta diversity, principal-coordinate analysis [PCoA], etc.). While these methods are useful for identifying the characteristics of a microbial community as a whole, the information about each individual taxon is often lost, and interesting behaviors of some microbial subgroups can be masked. Moreover, these methods can give rise to misleading results in certain cases. For example, the distances between microbial samples can plateau along a gradient of environmental variables and cause horseshoe effects on an ordination plot, leading to confusion in data interpretation (6, 7). As a complementary analysis approach, looking at each taxon individually may facilitate teasing apart the key players driving interesting community dynamics. However, it is challenging to observe or visualize large sample-by-feature tables. Doing so naively often obscures important patterns in the data. Careful data manipulation and convenient data exploration procedures are needed to reveal the dynamic patterns of each microorganism within a community.

There are existing tools for analyzing and visualizing an entire microbiome data set. For example, Calypso is a web application allowing nonexpert users to explore and compare taxonomic profiles from 16S or shotgun data (8). MEGAN provides a rich set of taxonomic and functional analyses with a graphical user interface (9). STAMP, with a focus on statistical hypothesis tests, also offers a graphical interface for users to study taxonomic and functional profiles (10). Nevertheless, no existing tool was able to meet our workflows’ particular needs for interactively examining specific features, including rapid filtering and sorting of both taxa and samples by multiple criteria (see Table S1 in the supplemental material). Here we introduce Calour, an interactive data exploration method based on heatmaps, to represent a microbiome data set without data reduction. This representation provides a first-hand understanding of the microbial trends in the study. Calour also provides a suite of data manipulation (filtering, sorting, clustering, and transforming) and statistical (discrete false-discovery rate [FDR] [11], correlation, and differential abundance) tools to facilitate fast data exploration and to identify subgroups of interesting microbes. Additionally, Calour can incorporate results from other statistical methods of choice through the utilization of feature metadata—which is critical given the rapid development of new differential abundance and correlation metrics. Visual inspection of the data can reveal interesting behaviors of microbes in an unsupervised fashion, because human eyes are very sensitive at pattern recognition. Those observations can lead to hypotheses that can then be rigorously tested using statistical methods or further experiments. Calour also enables automatic queries against knowledge databases of amplicon sequences. This feature empowers users to associate their study within a larger context and build upon what is already known about a particular taxon of interest. In this paper, we demonstrate how Calour can be used in five published data sets to identify microbial and metabolite patterns and develop novel biological hypotheses: the effect of habitat switching on the skin and gut microbiome of salamander larvae (12), the gut microbiome in Trichuris muris-infected mice (13), a cross-sectional study of skin microbiome (14), a low-biomass ant gut microbiome study (15), and a longitudinal metabolome study on the effects of intermittent hypoxia and hypercapnia and diet on fecal metabolites in mice (19).

10.1128/mSystems.00269-18.5 TABLE S1 Comparison of functionalities of Calour, MEGAN, STAMP, and Calypso. Download Table S1, DOCX file, 0.01 MB. Copyright © 2019 Xu et al. 2019 Xu et al. This content is distributed under the terms of the Creative Commons Attribution 4.0 International license.

## RESULTS AND DISCUSSION

### Case study 1: habitat switching in amphibian larvae.

**FIG 1:**
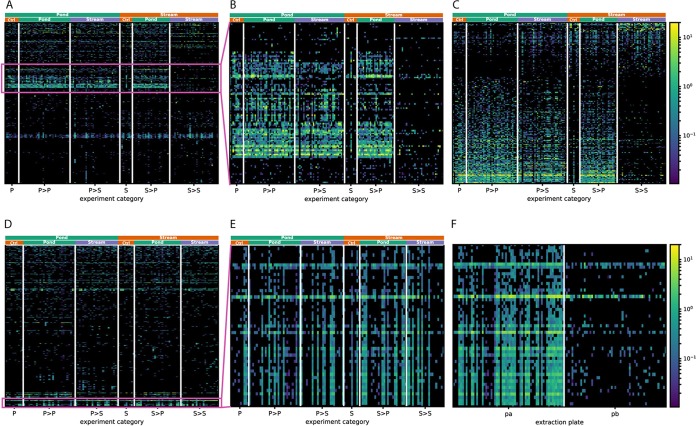
Exploration of gut and skin microbiome of the habitat switching experiment with Calour. Larvae originating from a pond (P) or stream (S) were transferred to a different environment or to the same environment (pond to stream [P>S], pond to pond [P>P], stream to pond [S>P], stream to stream [S>S]). Gut samples (A to C) and skin samples (D to F) were used. (A) An overview of all gut sOTUs with samples in columns and sOTUs in rows clustered by the similarity of their abundance profiles. The top color bars indicate the origin environments and the destination environments for each sample, including “Pond,” “Stream,” and “Ctrl” (control [for individuals staying in the origin environment without transferring]). This is similar for the following figure panels. The color scale is shown as a percentage. (B) A zoomed-in view of a group of gut sOTUs enriched in samples that were exposed to pond water at any time point (P, P>P, P>S, S>P). (C) Gut sOTUs significantly different in abundance between stream-only (S, S>S) and pond-only (P, P>P) groups and using permutation-based dsFDR. Note that the sOTU profiles of the P>S and S>P samples are similar to those of the P>P samples, confirming what we see in panel B. (D) An overview of all skin sOTUs. (E) A zoomed-in view of a correlated group of skin sOTUs from panel D. Their abundance pattern across samples is not explained by origin or destination environments. (F) After samples are sorted according to extraction plate, we see that the patterns can be explained by the extraction plate, with these sOTUs more abundant in plate pa than in plate pb.

In this study (12), fire salamander larvae originating from ponds (P) or streams (S) were either kept in the same environment (P→P, S→S) or transferred to the other environment (P→S, S→P) for 2 weeks, after which the skin and gut microbiome of the larvae was sampled and sequenced. Additional individuals naturally residing in the ponds (P) and streams (S) at the end of the 2-week experiment were also sampled and sequenced as controls. The data set was downloaded from SRA (accession no. PRJNA320968) and processed into an sOTU table with Deblur (see Materials and Methods for details). This biom table and the corresponding metadata file were imported into Calour, and all downstream processing was performed using Calour functions. Data were first normalized to 10,000 reads/sample using total sum scaling, and the low-abundance sOTU (total normalized reads over all samples < 10) were filtered away, resulting in skin and gut biom tables containing 1,650 and 975 sOTUs, respectively. To obtain an overview of the trends in the data set, we first sorted the samples by their origin and destination environments and clustered sOTUs based on their abundances across samples. The resulting heatmaps for gut and skin samples are shown in Fig. 1A and D, respectively. In Fig. 1A, we see a group of sOTUs enriched in samples that were in contact with the pond at any time point (P, P→P, P→S, S→P). This becomes more obvious when we interactively zoom in on the heatmap in Calour (Fig. 1B). This indicates that these pond-specific microbes are strong colonizers of the larvae’s gut and resistant to environmental change. In order to rigorously elucidate this pattern, we used a permutation-based nonparametric differential abundance rank mean test with a discrete false-discovery rate (dsFDR) (11) multiple-hypothesis correction implemented in Calour and applied this analysis between pond-only groups (P, P→P) and stream-only groups (S, S→S) on gut samples (Fig. 1C) and skin samples (see Fig. S1A in the supplemental material), respectively, to identify environment-specific sOTUs. This identified 267 and 113 sOTUs that have significant differences between pond-only and stream-only samples in gut and skin samples, respectively. Concurring with the visual inspection in Fig. 1B, 75 out of the 76 sOTUs present in the pond visual cluster are also among the 267 sOTUs detected by the differential abundance test. In summary, we can make the following observations. (i) There are more pond-specific sOTUs than stream-specific sOTUs in both the gut and skin. (ii) Pond-specific sOTUs have dominating colonization effects in the gut, as the P→S gut samples still had abundant pond sOTUs after 2 weeks in stream habitats (Fig. 1B and C). (iii) The skin microbiome does not have this dominating colonization property; instead, it reflects the current environment, as P→S skin samples are more similar to S and S→S groups, and S→P samples are more similar to P and P→P samples (Fig. S1A). We note that these findings are complementary to PCoA analyses. For example, both the weighted UniFrac PCoA (Fig. S1B) and the unweighted UniFrac (Fig. 4C in reference 12) show P→S samples lie in the middle between the P→P and S→S samples and lean toward P→P samples. Exploration with Calour provides direct visualization of how this pattern occurs and what specific groups of sOTUs are responsible for it.

10.1128/mSystems.00269-18.1 FIG S1 Microbiome of the transfer experiment. Larvae originating from a pond or stream (P or S, respectively) were transferred to a different or the same environment (P>S, P>P, S>P, and S>S indicate pond to stream, pond to pond, stream to pond, and stream to stream, respectively). (A) Skin bacteria significantly different between S and S>S and between P and P>P using permutation-based dsFDR. The S>P and P>S samples were not included in the differential abundance testing. Note that P>S bacteria are similar to S>S bacteria, while S>P bacteria are similar to P>P bacteria. (B) PCoA analysis of weighted UniFrac distance matrix of gut samples. The numbers in parentheses indicate the numbers of samples. Download FIG S1, TIF file, 0.2 MB. Copyright © 2019 Xu et al. 2019 Xu et al. This content is distributed under the terms of the Creative Commons Attribution 4.0 International license.

When interactively exploring the Calour heatmap, we observed a cluster of sOTUs in the skin samples whose abundances cannot be explained by the sample time point, origin or destination environment, or other provided sample metadata (Fig. 1E). After investigating additional sample processing information, we found that the pattern of this set of sOTUs can be explained very well by extraction plate, which becomes obvious after the samples in each group are sorted by extraction plate (Fig. 1F). These samples were processed in two different DNA extraction plates by two different individuals. One possible explanation is that these bacteria are extraction plate-dependent contaminants. Alternatively, since some of these bacteria are present in both plates but at different frequencies, these microbes could be more sensitive to the DNA extraction protocol used in the study and their abundance differences may result from different extraction efficiencies when plates were handled by two different individuals.

### Case study 2: chronic Trichuris muris infection in mice.

This study examined the effect of chronic infection with the helminth parasite Trichuris muris on the murine cecal microbiome (13). Mice were divided into two groups: one group was infected with Trichuris muris eggs at day 0, whereas the second group served as controls. At several time points, fecal samples were collected from each group, and the V4 region of the 16S rRNA gene was sequenced. The data were downloaded from NCBI SRA (accession no. PRJEB6560) and analyzed by Deblur to compute the sOTU table. The data were imported into Calour with sOTUs reordered by clustering (in rows) and samples sorted by time point and treatment (in columns). The Calour heatmap overview reveals that differences between infected and uninfected groups occurred mostly at days 27 and 35 postinfection (Fig. S2A), which was also observed in the original paper (13) and in the weighted UniFrac PCoA plot (Fig. S2B).

10.1128/mSystems.00269-18.2 FIG S2 Overview of all sOTUs in the mouse infection experiment. (A) All the sOTUs in the experiment after feature clustering and sample sorting. A difference can be observed between the control and the *T. muris*-infected groups on days 27 and 35. (B) PCoA plot based on weighted UniFrac distance. Each dot represents a sample colored by day. The numbers in parentheses are sample counts. Infected samples (large spheres) and uninfected samples (small spheres) are indicated. Download FIG S2, TIF file, 0.8 MB. Copyright © 2019 Xu et al. 2019 Xu et al. This content is distributed under the terms of the Creative Commons Attribution 4.0 International license.

**FIG 2:**
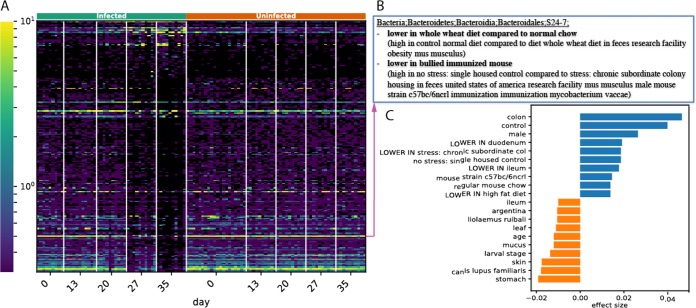
Calour analysis of mouse gut microbiome with chronic Trichuris muris infection. A group of mice was infected with Trichuris muris at time point 0 and compared to uninfected control mice. The colon samples were taken during a 35-day period. (A) sOTUs that pass the differential abundance test between uninfected mice and the infected mice on days 27 and 35. (B) An example of database annotations for a single sOTU higher in the uninfected mice than in the infected mice. (C) Top 10 significantly enriched terms found in the sOTUs higher in the uninfected controls (blue) or higher in the infected mice (orange).

We then did a differential abundance test to filter the sOTUs that differ between control and infected mice at days 27 and 35. As shown in Fig. 2A, there are a large number of sOTUs that are not detected at day 35, whereas a smaller number of sOTUs increase in relative abundance. When we click on a row (i.e., an sOTU) in the heatmap, the annotations associated with this particular sOTU (as in Fig. 2B) from various microbial databases will pop up. For this analysis, we focus on dbBact database annotations, which contain manually curated observations about sOTUs (such as different abundances in sick versus healthy people in a given study). In order to generate a hypothesis for this change, we used Calour enrichment analysis to find biological terms from the dbBact database that are enriched in the group of decreasing bacteria compared to the other increasing group (see Materials and Methods for details). Since databases such as dbBact are incomplete (i.e., do not contain all the known information about each bacterium) and may contain studies of varying quality, we use Calour to compare two groups of bacteria from the same experiment (i.e., decreasing versus increasing), obtaining a list of statistically significant enriched terms in either of the groups. The top terms associated with the bacteria that decreased in the infected group include “c57bl/6,” “LOWER IN colotis,” and “rat” (blue bars, Fig. 2C), whereas the ones associated with increasing bacteria include “skin,” “high fat diet,” “leaf” and “ocean.” This can lead to a possible biological hypothesis: following the hatching of Trichuris muris, there is a general stress response in the mouse gut, with possibly shorter bowel transit time, resulting in diluted colon-inhabiting microbes and more DNA from bacteria associated with other environments (like the stomach) reaching the feces.

### Case study 3: skin bacteria on various body sites.

**FIG 3:**
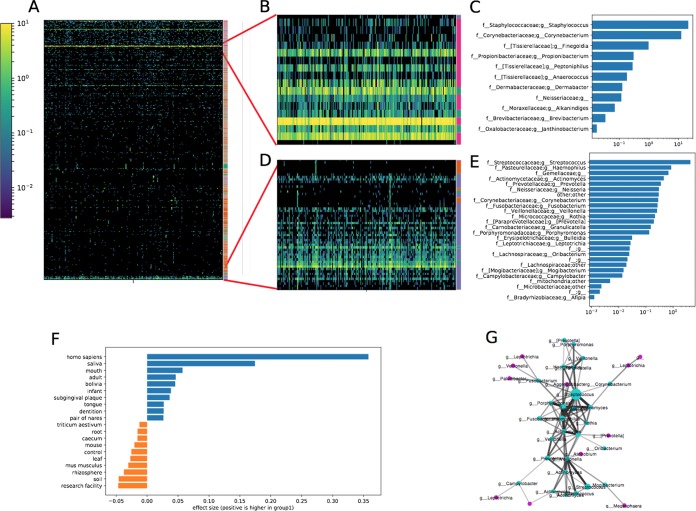
Analysis of skin samples from reference 11. (A) All sOTUs in arm samples, with sOTUs clustered by abundance. (B) A zoomed-in view of panel A showing a cluster with a large number of skin-associated sOTUs. The vertical color bar on the right indicates the most common dbBact annotation out of skin (pink), saliva (blue), feces (green), or other (orange). (C) Taxonomies of the sOTUs in the cluster shown in panel B and their abundances (collapsed at the genus level). (D and E) Similar to panels B and C but showing a cluster with a number of saliva-associated bacteria and their taxonomies. (F) Enriched terms for the cluster shown in panel D compared to the rest of the sOTUs. Blue bars indicate terms significantly enriched in this cluster of bacteria, orange bars show terms enriched in the rest of the skin bacteria (dsFDR controlled at 0.1). (G) A cooccurrence network confirms the presence of the correlated sOTU cluster that is associated with saliva annotation term. The darkness and width indicate the correlation strength inferred by local similarity analysis (14, 15, 17). The size of the node represents the abundance of each sOTU. The sOTUs in the cluster shown in panel D are shown in cyan, and all the other sOTUs are shown in magenta.

This study measured the skin microbiome in male individuals from New York City, New York, at two time points (14). Its raw data were obtained from NCBI SRA (accession no. PRJNA314604). After clustering sOTUs based on their abundance profiles, several groups of similarly behaving sOTUs are visible in arm samples (Fig. 3A). When interactively looking at sOTUs from these clusters, annotations from dbBact indicate some clusters contain microbes often associated with one of the following annotations: skin, saliva, or feces. We therefore add a vertical color bar indicating the most common term in dbBact (out of “feces,” “saliva,” and “skin,” colored with green, blue, and pink, respectively) for each sOTU in the two most striking clusters (Fig. 3B and D). The cluster in Fig. 3B contains a large number of bacteria with dbBact annotations associated with skin, such as *Staphylococcus* and *Corynebacterium* (Fig. 3C), whereas the cluster in Fig. 3D contains bacteria that have mostly saliva dbBact annotations. We performed dbBact term enrichment analysis in this cluster compared to the rest of the sOTUs in this experiment. As shown in Fig. 3F, the cluster is significantly enriched in mouth-associated annotation terms from dbBact, including saliva, mouth, subgingival plaque, and tongue. This saliva sOTU cluster is made of sOTUs from a large variety of taxonomies (Fig. 3E) that are correlated across different individuals, as independently confirmed by cooccurrence analysis shown in Fig. 3G. A similar saliva cluster is also observed in the skin samples from the American Gut Project (17) (Fig. S3). Intriguingly, the majority of these saliva sOTUs are also reported to form organized, complex biofilm structures in dental plaque (16). This leads us to conjecture that these bacteria may tightly interact with each other (possibly due to the molecular properties of their membranes) and thus, have the propensity to assemble to a biofilm in oral cavity and on skin.

10.1128/mSystems.00269-18.3 FIG S3 Analysis of skin samples from the American Gut Project. Analysis is based on the December 2016 release (ftp://ftp.microbio.me/AmericanGut). (A) All sOTUs from American Gut Project skin samples, with sOTUs clustered by abundance. (B and C) Zoomed-in views of panel A showing clusters, with vertical color bar indicating the most common dbBact annotation out of skin, saliva, and feces (blue, pink, and orange, respectively). The clusters shown in panels B and C are most associated with skin and saliva, respectively. (D) Enriched terms for the cluster shown in panel C compared to the rest of the skin bacteria. Blue bars indicate terms significantly enriched in the clustered bacteria, orange bars show terms enriched in the rest of the skin bacteria (dsFDR controlled at 0.1.) (E) Taxonomies of saliva-related cluster shown in panel C and their abundances (collapsed at the genus level). Download FIG S3, TIF file, 1.4 MB. Copyright © 2019 Xu et al. 2019 Xu et al. This content is distributed under the terms of the Creative Commons Attribution 4.0 International license.

### Case study 4: identification of contaminants and host-specific bacteria in low-biomass ant experiment.

**FIG 4:**
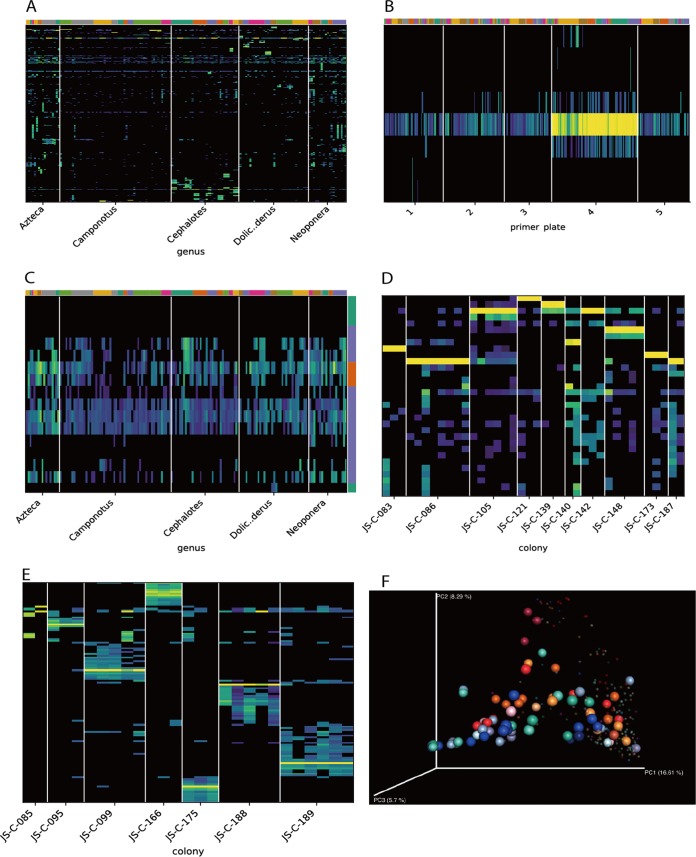
Analyses of low-biomass ant gut microbiota. (A) Distribution of sOTUs across samples sorted by ant genus and colony. The top color bars indicate the ant colony of each sample. (B) A zoomed-in view showing the DNA extraction plate-dependent abundance profile of Acinetobacter johnsonii after the samples were sorted by plate. (C) A zoomed-in view of panel A showing a group of bacteria present in all samples across all ant genera. The color bar to the right indicates whether each sOTU is a known contaminant (orange) or has other annotations (blue) or no annotations (green) based on dbBact annotations. (D and E) Colony-specific abundance profiles of sOTUs within ant genera of *Camponotus* (D) and *Dolichonderus* (E) following filtering of contaminants and re normalization of the samples. Only sOTUs present in at least 10% of the samples are shown. (F) Unweighted UniFrac PCoA of all ant samples. *Camponotus* and *Dolichoderus* samples are colored by colony; all the other samples are in small and transparent spheres.

This experiment involved sequencing of various ant species from Peru and studying the relationship between diet, habitat, and bacterial biomass (15). Using Calour to interactively examine the sOTU abundances (Fig. 4A), we can see genus- or colony-specific sOTUs as well as some widespread sOTUs. In order to look for potential contaminants (since these are low-biomass samples), we examine these widespread sOTUs more specifically. As shown in Fig. 4B, an sOTU (Acinetobacter johnsonii) shows a plate-dependent distribution, with very high abundances (>90% of the reads) in almost all plate 4 samples and nonzero abundances in virtually every other sample. Although samples in plate 4 were taken from ants at a different life stage from the samples in the other plates, A. johnsonii was not detected in a previous survey of a subset of these ant species (18), suggesting that it may be a chance (if unusual) contaminant of this sample set. Another candidate group of contaminants is a cluster of widespread bacteria with uniform distribution profiles (Fig. 4C). In order to assess whether these may be derived from reagent contamination, we looked at whether any of these sequences are known contaminants in other experiments as annotated in dbBact. It turns out that two of the bacteria in this cluster (indicated in orange in the vertical color bar in Fig. 4C) have been observed as contaminants in other experiments, consistent with an origin in reagents rather than biological samples. After removing these contaminants and renormalizing the samples using Calour, we can see a strong colony dependence in the bacterial composition within a single genus for both *Camponotus* (Fig. 4D) and *Dolichonderus* (Fig. 4E), where many abundant sOTUs are either present at a very high level or not present at all, depending on the colony. In *Camponotus*, each colony (most of which belong to distinct species) is dominated by its own type of *Blochmannia*, a known endosymbiont of this genus. A similar pattern is observed for *Dolichoderus*, which appear to host undescribed colony-specific lineages of *Bartonella*-related bacteria, consistent with microscopic evidence from the primary publication (15). Unlike *Camponotus*, however, the *Dolichoderus* also appear to host a complement of other colony-specific bacteria, suggesting that there may be deeper underlying differences in the relationship between host and bacteria in these two arboreal ant genera. These discrete sample/sOTU patterns, clearly apparent in the interactive heatmaps in Calour, are not apparent in distance-based sample ordinations (Fig. 4F), highlighting the utility of the heatmap-based visualization as a tool for data exploration.

### Case study 5: using Calour for mass-spec data analysis—effect of intermittent hypoxia and hypercapnia and diet on fecal metabolites (19).

Calour can also be used for mass-spec experiment analysis, combining information from the Global Natural Products Social Networking (GNPS) metabolomics database (20). We analyzed longitudinal metabolite data from the feces of mice undergoing intermittent hypoxia and hypercapnia (IHH) compared to controls. After sorting by time point and treatment group, we can see the largest effect on the metabolites in this experiment happens after week 10 (Fig. S4A). This corresponds to the switch from normal mouse chow to a Western, high-fat diet. Filtering only metabolites with GNPS curations (using the GNPS database interface module), we keep 653 out of the 1,099 metabolites. We then applied the dsFDR-corrected differential abundance test (for presence/absence) (11) and found 48 and 164 metabolites associated with mouse chow and Western diet, respectively (Fig. S4B and Table S2). Examining GNPS-derived curations of these metabolites indicates that as mice are switched to a high-fat diet, there is a large increase in bile acids in the gut metabolome, derived from both the host (e.g., taurocholic acid and cholic acid) and microbe (e.g., deoxycholic acid, tauroursodeoxycholic acid, and 12-ketodeoxycholic acid), as reported by previous studies as well (21, 22).

10.1128/mSystems.00269-18.4 FIG S4 Mass-spec data analysis. Examining the effect of intermittent hypoxia and hypercapnia (IHH) and diet on fecal metabolites. (A) All metabolites from the experiment. Rows and columns represent metabolites and samples, respectively. Samples are divided according to the treatment group (control [air] and hypoxia [IHH]) and sorted by the sampling week (shown in the top bar label). (B) Focusing only on metabolites with known GNPS names, which are diet related (those different between week 10 and the rest of the samples). Metabolites were identified using a presence/absence permutation test with dsFDR controlled at 0.1. A full list of metabolite names is available in Table S2 in the supplemental material. Due to the large number of metabolites identified, the figure shows GNPS names every few metabolites on the *y* axis. (C) Metabolites with known GNPS names that are different between control and hypoxia groups. After removal of the week 10 samples, metabolites were identified using a presence/absence permutation test with the dsFDR controlled at 0.1. A full list of metabolite names is available in Table S3. (D) Ordered PCoA of air (red) and IHH (blue) samples based on Bray-Curtis distance of metabolites. Samples are sorted on the first axis by age (left to right), with the two main PCoA axes explaining 34% and 12% of total sample variance. Download FIG S4, TIF file, 2.9 MB. Copyright © 2019 Xu et al. 2019 Xu et al. This content is distributed under the terms of the Creative Commons Attribution 4.0 International license.

10.1128/mSystems.00269-18.6 TABLE S2 The metabolites that are differentially abundant between mice fed a normal diet and high-fat diet in case study 5. Download Table S2, XLSX file, 0.01 MB. Copyright © 2019 Xu et al. 2019 Xu et al. This content is distributed under the terms of the Creative Commons Attribution 4.0 International license.

After removing the samples prior to diet switch and applying the presence/absence differential abundance test between IHH and control groups, we identified 16 and 22 metabolites associated with the IHH and control groups, respectively (Fig. S4C and Table S3). These metabolites contain a large number of bile acids (cholic acid, taurocholic acid, chenodeoxycholic acid, tauroursocholic acid, etc.) and hormones (5-androstene-3β,16α,17α-triol, 5β-pregnane-3α,17-diol-20-one) among other molecules. Hence, we can hypothesize that the downstream effects of IHH could be linked to alterations in bile acid pool and endocrine disruption. Note that these are spectral alignment-based annotations (level 2 annotations) according to metabolomics reporting standards (20, 23) and should be confirmed by comparison with pure analytical standards (level 1 annotations). This difference between the IHH and control groups is less evident when examining the PCoA of the samples (Fig. S4D).

10.1128/mSystems.00269-18.7 TABLE S3 The metabolites that are differentially abundant between control group and IHH (intermittent hypoxia and hypercapnia) group in case study 5. Download Table S3, XLSX file, 0.01 MB. Copyright © 2019 Xu et al. 2019 Xu et al. This content is distributed under the terms of the Creative Commons Attribution 4.0 International license.

### Conclusion.

We introduced Calour as a tool for interactive exploration of microbiome data sets. With multiple user interfaces, Calour is intended to be useful to both experts and nonexperts as part of an overall microbiome analysis workflow. Specifically, the feature-level analysis of the data in Calour provides complementary insights to alpha and beta diversity measures and ordination plots, which work at the whole-community level. The visualizations provided by Calour are a complement to feature-level statistical and machine learning analyses such as ANCOM or random forests feature selection, which identify which features are important for separating groups of samples but do not provide a direct way of visualizing the distribution of those features across samples and revealing subtle microbial patterns. Calour enables this important part of the workflow, both early in exploratory analysis and at the end when proper filtering, clustering, and sorting are required to produce publication-ready figures. Its integration with microbial annotation databases further empowers users to incorporate known information about each microbe present in the experiment. These advantages of Calour allow users to generate novel biological hypotheses, which can then be validated with further analyses and targeted experiments.

## MATERIALS AND METHODS

### Calour implementation.

**FIG 5:**
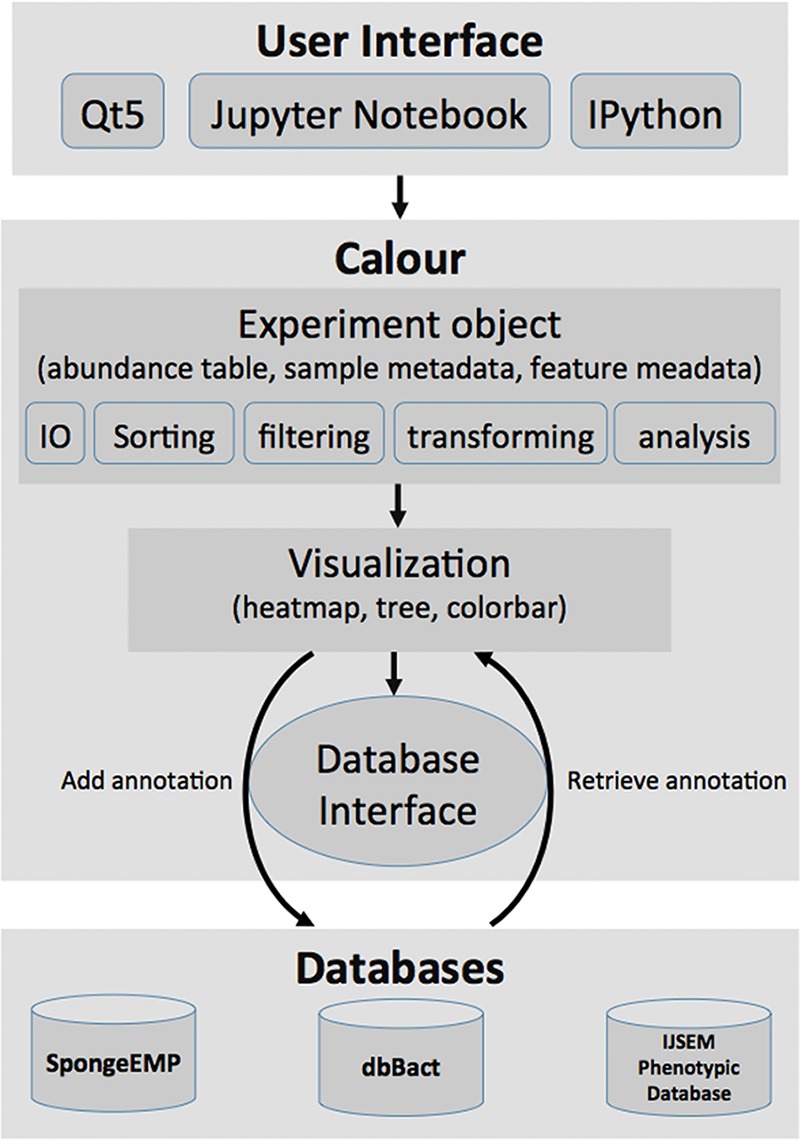
Implementation structure of Calour. Calour can be interfaced with Qt5 GUI, Jupyter Notebook, or IPython interactive session. All the functionalities in the modules (input/output [IO], sorting, filtering, data transforming, machine learning, and statistical analyses) operate on the core object, the Experiment object, which integrates the abundance table, sample metadata, and feature metadata together. A heatmap can be rendered to interact with databases to add or retrieve annotation. All the operations of the Experiment object are recorded by Calour so that the process is tractable and reproducible.

Calour can be used either through Python or Jupyter Notebook interface, or through a GUI (graphical user interface) based on Qt5 (Fig. 5), allowing users without bioinformatics expertise to explore their data. The software is implemented in Python 3 and runs on Windows, Mac OSX, and Linux platform. The code is unit tested, with detailed Application Programming Interface (API) documentation and tutorials (http://biocore.github.io/calour/).

Calour utilizes a study-centric data model integrating the 2D abundance table, sample metadata, and feature metadata. Every procedure provided by Calour operates on this data model and synchronizes the abundance table and its metadata. It depends on the widely used packages, including numpy, scipy, pandas, biom (24) and scikit-bio. Calour includes the following modules (Fig. 5).

**(i) IO (input/output) module.** The input/output (IO) module handles importing and exporting of tabulated data and metadata. It currently supports biom tables, tab-delimited text tables, and metabolomics MS1 bucket tables for abundance data, and tab-delimited metadata files.

**(ii) Experiment module.** The experiment module defines the main Calour object class, which contains sparse or dense 2D abundance tables and links them to per-feature and per-sample pandas DataFrame objects of metadata. Additionally, the experiment class stores the history of all the operations that have been run so that the whole exploratory process is tractable afterwards. Child classes are defined for specific experiment types (e.g., amplicon experiment and metabolomics experiment).

**(iii) Transforming module.** The transforming module deals with modifications of the abundance table via normalization and data transformations. Normalization methods include total sum scaling, total sum scaling with removal of outliers, and center log ratio transformation. Data transformations include binarization and log transformation (using a minimal read cutoff to handle zeros in the data).

**(iv) Sorting module.** The sorting module provides the functions to reorder either samples or features by any arbitrary functions (e.g., the abundance or prevalence of the features or associated metadata). It also provides a function for sorting the features by their center of mass across a fixed set of samples. The sample/feature clustering function is also implemented in the sorting module, with defaults set to single linkage hierarchical clustering following log transformation and per-feature centering and unit variance normalization.

**(v) Filtering module.** The filtering module provides functions for filtering either samples or features by data or metadata criteria (such as minimal abundance/prevalence, specific metadata values, etc.)

**(vi) Analysis module.** The analysis module implements statistical tests for finding associations between data table and metadata. It includes FDR-controlled permutation-based nonparametric tests for differential abundance and correlation. The differential abundance tests compare a per-feature statistic between two sample groups after an optional data transformation such as ranking and binarizing. The same statistic is computed many times, each time after a random group label permutation. Then the original statistic is compared to the distribution of the statistic from random permutations to compute *P* values. For correlation analysis, a similar approach is used for correlating a continuous metadata field to feature prevalence, using Spearman, Pearson, or other user-defined correlation metrics. All tests include Benjamini-Hochberg (25), Gilbert’s filtered FDR (26), or dsFDR (11) for multiple hypothesis testing correction. With the convenient data model implemented in the Experiment object, Calour is readily extensible to incorporate additional statistical methods.

**(vii) Database module.** The database module defines the API to interact with a database to retrieve or add additional information about features. Currently, interfaces include the following.

*(a) spongeEMP.* spongeEMP (4) (http://www.spongeemp.com/main) is an automatically generated database for sequences found in the Earth Microbiome sea sponge samples. It provides per-sequence enrichment information about location/host/sample type as well as total prevalence in sponge samples.

*(b) IJSEM phenotypic database.* The IJSEM phenotypic database (5) is a manually annotated database containing phenotypes (such as growth temperature, pH, salinity preference, motility, etc.) of cultured bacteria.

*(c) GNPS.* GNPS (http://gnps.ucsd.edu) (20) is a metabolomics database that includes crowd-sourced MS/MS spectrum curation and automated molecular network analysis. Using the GNPS output file for the metabolomics table, the GNPS Calour interface allows integrating metabolite identification into the Calour-based mass-spec analysis.

*(d) dbBact.* dbBact (http://dbbact.org/main) is a manually curated annotation database of ontology-based observations derived from amplicon experiments. Observations include differential presence (e.g., bacterial sequence X is high in Homo sapiens with disease Y compared to controls), prevalence information (e.g., bacterial sequence X is common in habitat Y), as well as candidate contaminants (e.g., bacterial sequence X is a candidate contaminant in experiment Y).

Some database interfaces (e.g., dbBact) also enable term enrichment analysis between two feature groups. This is done by counting the number of times each term appears in both groups (with scoring based on the annotation type: 2 for “high frequency” or “higher in,” 1 for “common,” and −2 for “lower in”), and then applying rank mean test with dsFDR correction on all terms tested.

**(viii) Machine learning module.** The machine learning module contains functions to run classification and regression using scikit-learn library. These functions include random forest, linear regression, supporting vector machine, K nearest neighbors, etc. Users can check the scikit-learn website (https://scikit-learn.org/stable/) for a comprehensive list of methods available. Calour can also take other supervised classification or regression methods as long as they follow scikit-learn’s Application Programming Interface (API), including but not limited to XGBoost and Keras. It saves boiler-plate code for users to set up input and output for model training. The commonly used scoring function and plotting functions are also implemented. Specifically, visualization is available for confusion matrix and ROC curve for classification and scatter plot for regression. The notebook tutorial is available (https://biocore.github.io/calour/notebooks/microbiome_machine_learning.html).

**(ix) Heatmap module.** The heatmap module contains all the functions and classes to render a sophisticated heatmap and enable interactivity using a mouse and keyboard. Users can easily navigate through the whole heatmap by zooming and scrolling. For different user interfaces of Python, Jupyter notebook, and Qt5, the heatmap is rendered accordingly. In Jupyter Notebook, we take advantage of IPython Widgets to enhance the interactivity.

### Bioinformatic analysis.

The raw sequence data for all experiments were processed with Deblur (1) to generate an sOTU abundance table using default parameters. Taxonomies for the sOTUs were assigned using QIIME 1.9 (27) assign_taxonomy.py command and the RDP method using default parameters. Weighted and unweighted UniFrac distances were computed with the QIIME 1.9 pipeline and visualized in PCoA plots using Emperor (28). The cooccurrence network was inferred by local similarity analysis (29, 30). The network was rendered with Cytoscape v3.5 (31, 32) using “organic” layout.

### Data availability.

All data sets used in the paper are available publicly as described in each case study section. Jupyter Notebooks detailing the operations used for each analysis are available at https://github.com/knightlab-analyses/calour-manuscript for users to reproduce the results. Calour is also able to export heatmaps to an interactive html file to visualize the result without installation. The html files for the figures discussed in this paper are also available in this repository. Additional detailed Jupyter notebook tutorials are available for using Calour for microbiome and metabolomics analysis (http://biocore.github.io/calour/). A video tutorial for EZCalour (the full GUI for Calour) is available at https://www.youtube.com/watch?v=JQATqcgm31I, demonstrating the use of Calour for users without python knowledge.

Calour can be installed on Mac, Linux, and Windows, run as a VirtualBox image, or run without installing on a mybinder server (mybinder.org). Installation instructions covering these methods are detailed at https://github.com/biocore/calour/.
