# Supplementary material for: Calour: an Interactive, Microbe-Centric Analysis Tool
Source: mSystems. 2019 Jan 29;4(1):e00269-18. doi: 10.1128/mSystems.00269-18 (PMC6351725; doi:10.1128/mSystems.00269-18)
Supplement: TABLE S1 [file mSystems.00269-18-st001.docx]

Table S1. Comparison of functionalities of Calour, MEGAN, STAMP, and Calypso.

| **Features** | **Calour** | **MEGAN** | **STAMP** | **Calypso** |
| --- | --- | --- | --- | --- |
| **Main uses** | **Interactive and exploratory analysis and visualization of sample-by-feature data** | **Analysis of metagenome and metatranscriptome data** | **Analysis of taxonomic or metabolic profiles for statistical hypothesis tests** | **Analysis of taxonomic profiles from 16S amplicon or shotgun datasets** |
| **User interface** | **GUI, Python API, Jupyter Notebook API** | **GUI, CLI** | **GUI** | **Web-based GUI** |
| **OS platform** | **Linux, Mac, Windows (via virtualbox)** | **Linux, Mac, Windows** | **Linux, Mac, Windows** | **NA** |
| **Visualization** | **Interactive heatmap, barplots, scatter plots, other traditional displays.** | **PCoA (including biplots and triplots), trees, hierarchical clustering, heatmap, correlation networks** | **bar chart, PCA, boxplot, heatmap** | **bubbleplots, interactive hierarchical trees, Krona plots, heatmap** |
| **Data processing** | **sorting, normalization, filtering** | **NA** | **filtering** | **normalization** |
| **Statistical analysis** | **differential abundance, clustering, regression, classification** | **clustering** | **differential abundance, clustering** | **differential abundance, clustering, regression, classification,**  **network analysis** |
| **Database interface** | **dbBact, GNPS, IJSEM bacterial phenotype database, SpongeEMP*** | **KEGG, eggNOG, InterPro, SEED** | **NA** | **NA** |

* Database details:

**dbBact:** [dbbact.org](http://dbbact.org/main);

**IJSEM phenotypic database**: [github.com/amnona/pheno-calour](https://github.com/amnona/pheno-calour) (Barberán *et al.* Hiding in Plain Sight: Mining Bacterial Species Records for Phenotypic Trait Information. (2017) mSphere 2:e00237-17)

**SpongeEMP:** [www.spongeemp.com](http://www.spongeemp.com/main) (Moitinho-Silva *et al.* The sponge microbiome project. (2017) GigaScience 6:1-7)

**GNPS:** [gnps.ucsd.edu](https://gnps.ucsd.edu) (Wang M *et al.* Sharing and community curation of mass spectrometry data with Global Natural Products Social Molecular Networking. (2016) Nature Biotechnology 34:828–837)
