# Supplementary figures and images for: Calour: an Interactive, Microbe-Centric Analysis Tool
Source: mSystems. 2019 Jan 29;4(1):e00269-18. doi: 10.1128/mSystems.00269-18 (PMC6351725; doi:10.1128/mSystems.00269-18)

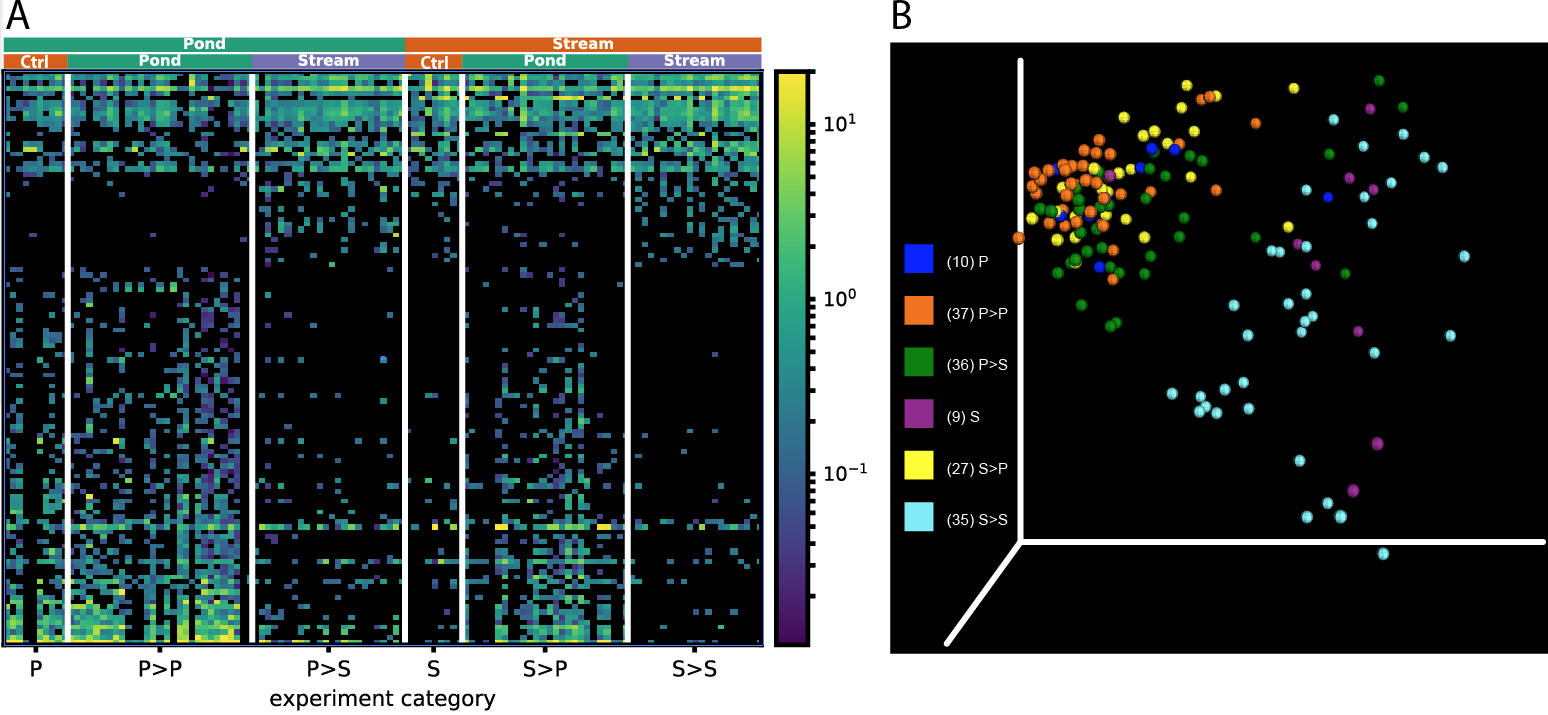

Supplement: FIG S1 [file mSystems.00269-18-sf001.tif]

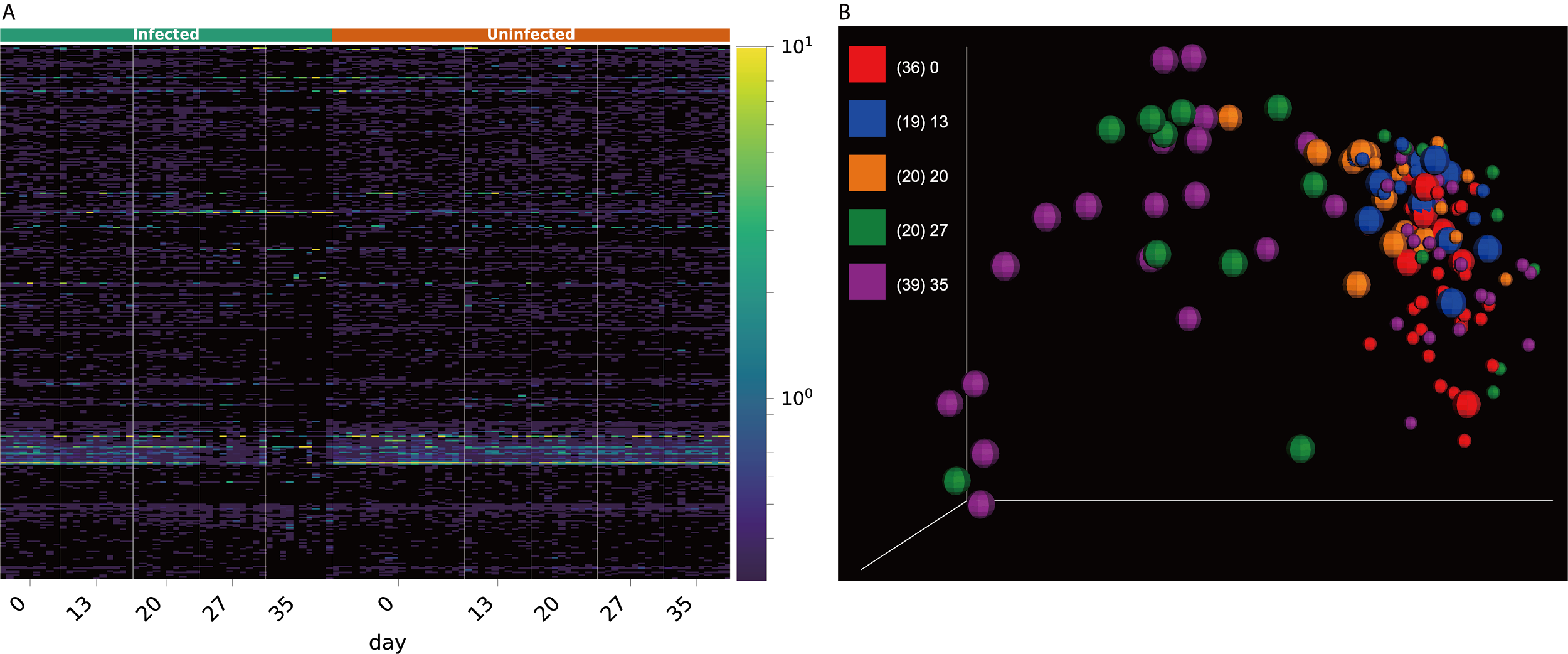

Supplement: FIG S2 [file mSystems.00269-18-sf002.tif]

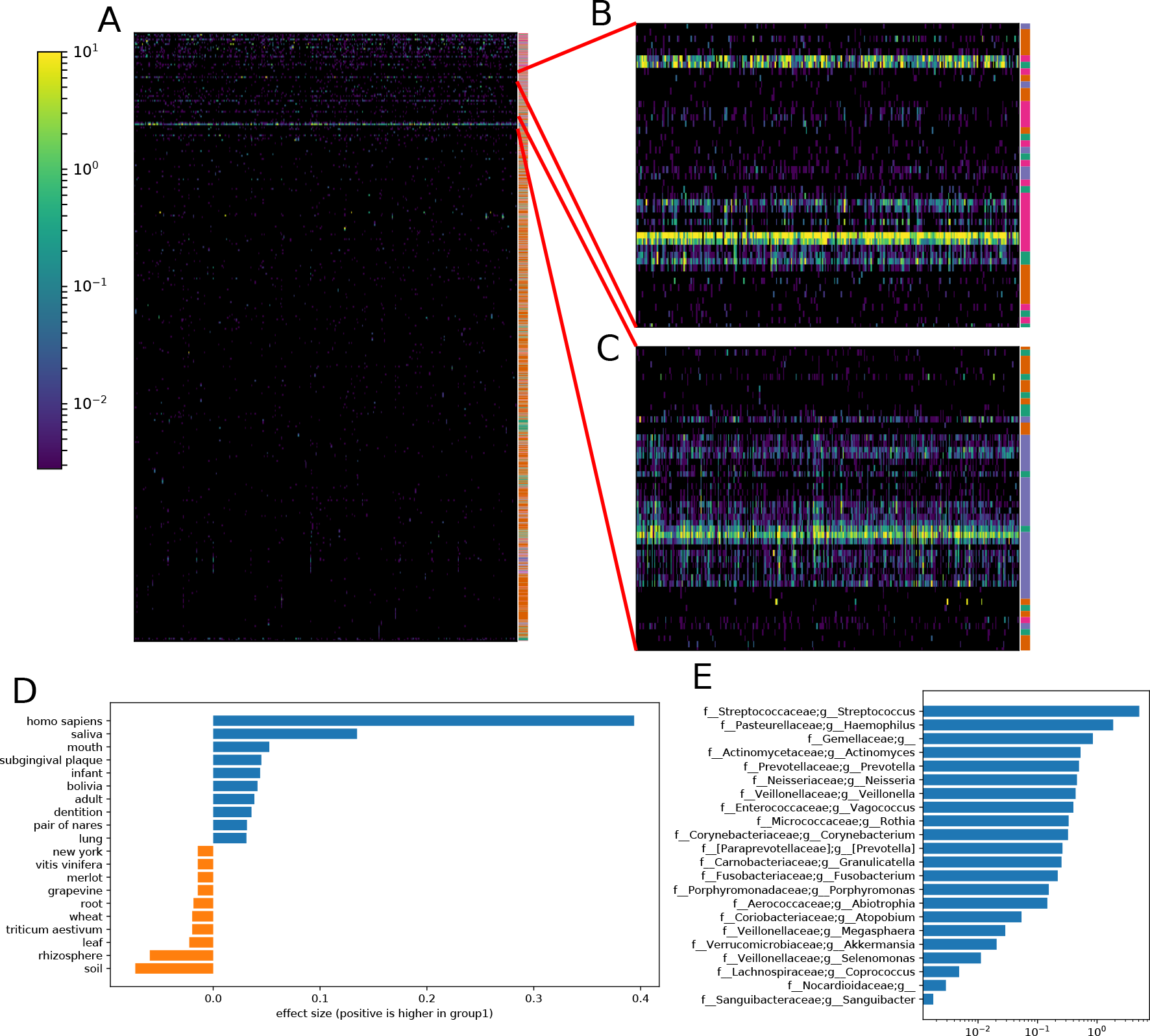

Supplement: FIG S3 [file mSystems.00269-18-sf003.tif]

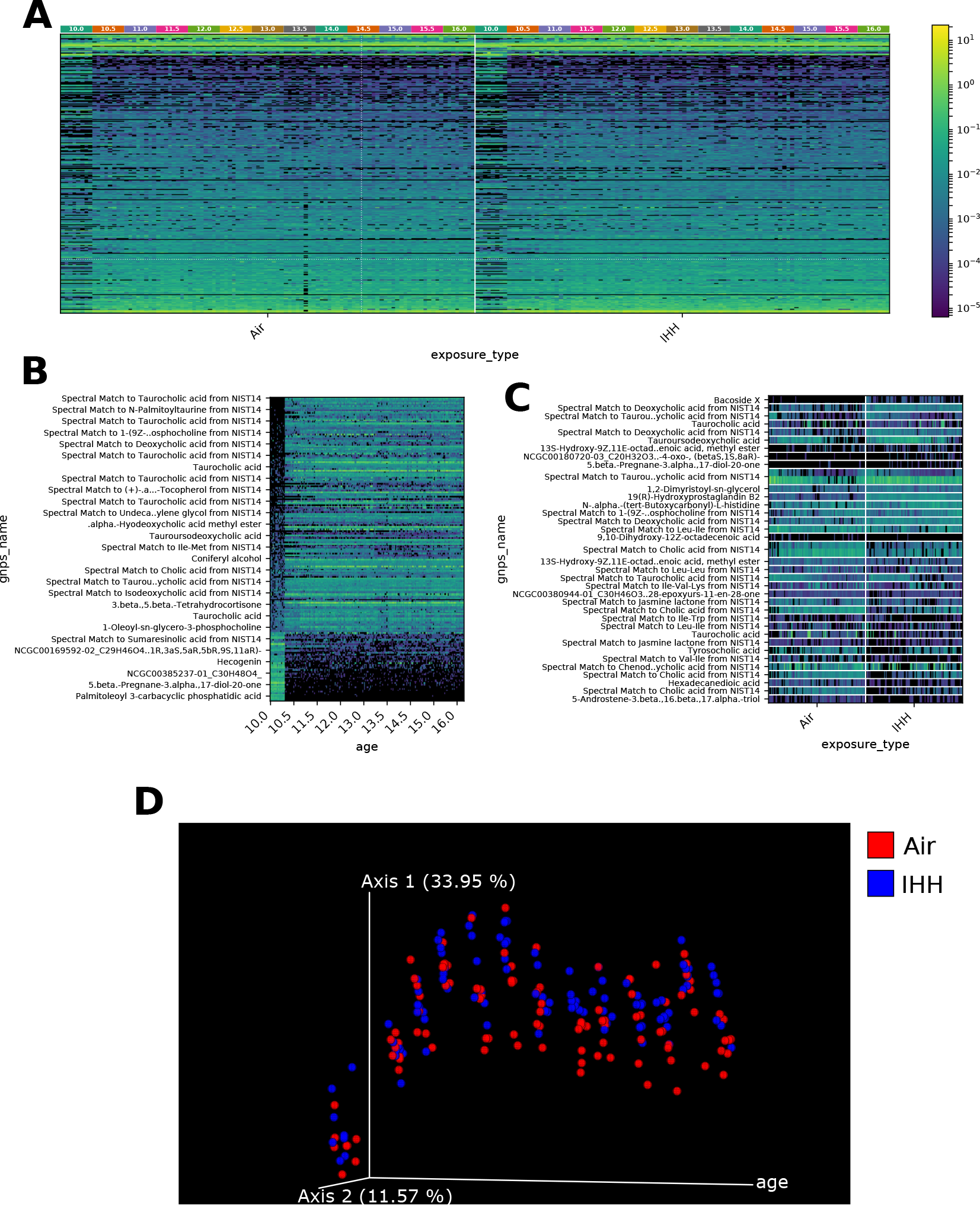

Supplement: FIG S4 [file mSystems.00269-18-sf004.tif]
